# The role of new synthetic membranes in rotator cuff augmentation

**DOI:** 10.1002/jeo2.70634

**Published:** 2026-01-21

**Authors:** Miguel Ángel Ruiz Iban, María Josefa Espejo Reina, Cristina Delgado del Caño, Salvador Álvarez Villar, Jorge Díaz Heredia, Jose Luis Ávila Lafuente

**Affiliations:** ^1^ Hospital Ramón y Cajal, Shoulder and Elbow Unit Madrid Spain; ^2^ Departamento de Cirugía, Ciencias Sanitarias Y Medicosociales, Alcalá de Henares, Facultad de Medicina Universidad de Alcalá Madrid Spain; ^3^ Fundación Jiménez Díaz, Shoulder and Elbow Unit Madrid Spain; ^4^ Hospital MAZ, Shoulder and Elbow Unit Zaragoza Spain

**Keywords:** bioinductive collagen implant, biological augmentation, rotator cuff, rotator cuff repair, shoulder

## Abstract

Rotator cuff tears (RCTs) are a highly prevalent source of pain and disability. Despite advances in surgical repair techniques, high retear rates remain concerning. Biological augmentation using synthetic membranes has emerged as a promising strategy to enhance tendon regeneration and reduce the risk of repair failure. The purpose of this study is to present the currently available evidence regarding new bioinductive membranes. A narrative review of the literature was performed to identify commercially available bioinductive membranes for rotator cuff augmentation and to summarize the evidence supporting their use. Currently, there are four implants commercially available: Regeneten® (Smith & Nephew, Biobrace® (Conmed), Tapestry® (Zimmer Biomet) and Integrity® (Anika Therapeutics). These scaffolds act as bioinductive matrices, promoting new tendon‐like tissue formation, intending to support the healing process and enhance the quality of the degenerative tendon, and reducing the risk of both failures at the tendon‐to‐bone interface and the occurrence of medial retears adjacent to the repair construct. All devices can be placed arthroscopically, although their fixation methods differ. Regeneten® has the most robust published evidence, whereas data on Biobrace® and Integrity® evidence remain limited, and no published outcomes are available for Tapestry®. Although complications are uncommon, cases of stiffness, inflammation and implant migration have been reported. Biologic augmentation with the new synthetic membranes is a promising technology that requires further attention. The use of the Regeneten implant seems to provide functional improvements in patients with partial tears and helps to avoid retears in full‐thickness RCTs. The evidence for the other membranes is limited at best today.

**Level of Evidence:**

Level V, expert opinion.

## Graphical abstract

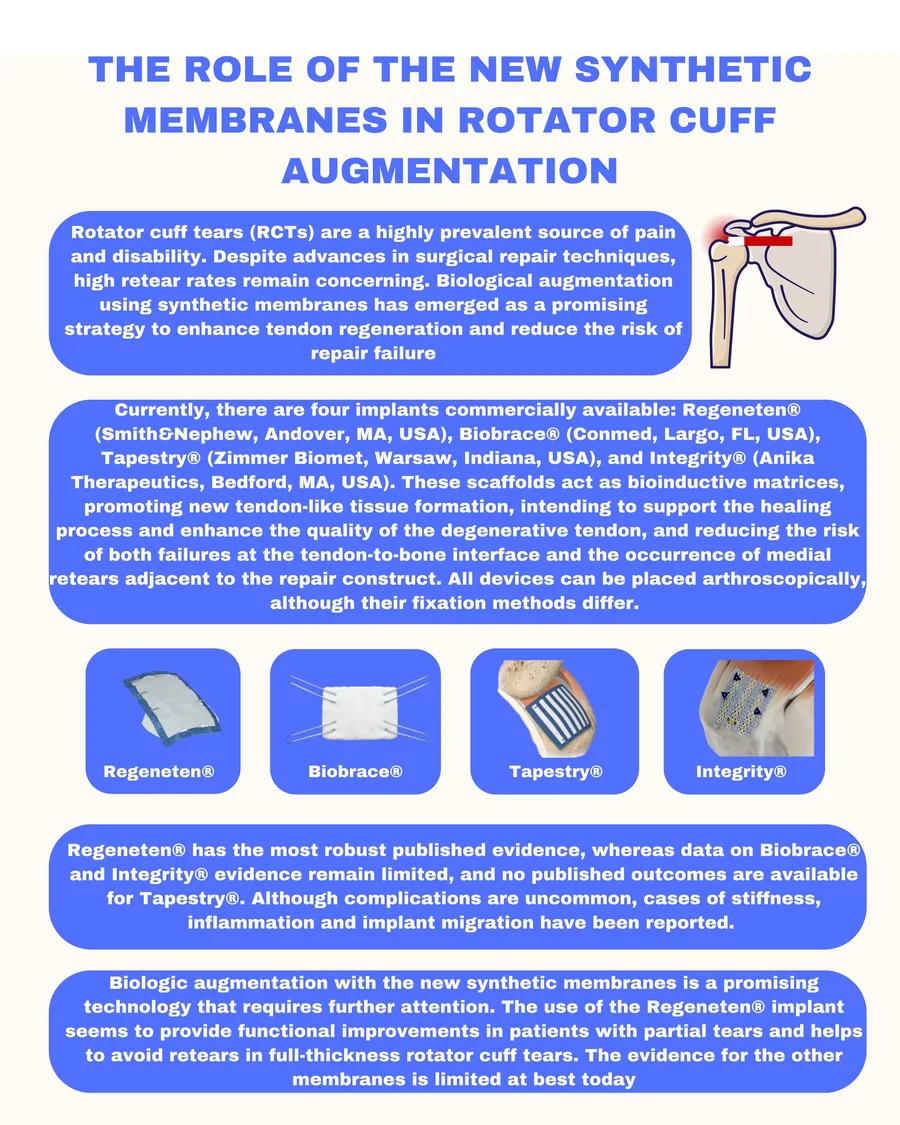

**Abbreviations**
FT‐RCT: full‐thickness rotator cuff tear
MCID: minimal clinically important difference
MRI: magnetic resonance imaging
PEEK: polyether ether ketone
PET: polyethylene terephthalate
PLGA: poly‐lactic‐co‐glycolic acid
PLLA: poly L‐Lactide
PRP: platelet‐rich plasma
PT‐RCT: partial‐thickness rotator cuff tear
RCR: rotator cuff repair
RCT: rotator cuff tear
TOE: transosseous equivalent

## INTRODUCTION

Rotator cuff tears (RCTs) are a recognised source of disability and psychosocial distress [25]. Between 16% and 34% of the general population is affected by RCTs [19]. The prevalence of this debilitating condition is higher among individuals older than 50 years, affecting up to 50% of this population [33]. Most RCTs are caused by degenerative rotator cuff disease, which is characterised by initial tendinopathy that eventually progresses to partial‐thickness tears (PT‐RCTs), and then to full‐thickness tears (FT‐RCTs); finally, rotator cuff arthropathy develops [12].

The treatment for PT‐RCTs is not yet well stablished. Typically, tears involving less than 50% of the tendon thickness are managed conservatively through activity modification and rehabilitation. If pain and dysfunction persist, surgical intervention can be considered [41]. Asymptomatic tears affecting more than 50% of the tendon generally do not require surgical treatment but should be monitored periodically with magnetic resonance imaging (MRI) [34]. In contrast, symptomatic tears involving more than 50% of the tendon thickness often require a surgical approach [22] including tear debridement, in situ transtendon repair or completion and repair of the tear [41]. However, retear rates following PT‐RCTs repair can reach up to 29% [27].

Arthroscopic rotator cuff repair (RCR) is the preferred surgical approach when conservative treatment fails in cases of FT‐RCTs, although most of these tears are initially treated nonoperatively. It is estimated that approximately 450,000 RCRs are performed every year in the United States [42]. Favourable outcomes have been reported following arthroscopic repair in both short‐ and long‐term follow‐up [40]. Nevertheless, high retear rates remain a significant concern, with reported rates ranging from 13.1% to 79%, depending on the study population and methodology [23]. Multiple factors have been identified as contributors to retear risk, including tendon quality, tear size and patient age. These variables are associated with a compromised biological environment, which impairs tissue healing and limits the restoration of the tendon's structural and functional integrity [18, 32].

Mechanical and biological factors are essential for successful rotator cuff healing. Despite significant advances in surgical techniques, improvements in healing rates remain limited [28]. From a mechanical perspective, sustained contact between the tendon and the bone for a sufficient duration is necessary for proper healing to develop. Biologically, tendon‐to‐bone healing is challenging and failures at the footprint (so‐called Cho type 1 failures [14] can occur. However, the most common failure pattern in contemporary repair strategies involves the tendon itself, which is usually degenerated, medial to the repair sutures (Cho type 2 failure) [14]. Various biological strategies have been proposed to enhance healing in RCTs, including the application of platelet‐rich plasma (PRP), stem cells, and the use of biological scaffolds and membranes of different materials [45]. The use of these new synthetic membranes with biological properties is a promising approach. Those new devices, when placed above the repaired tendon, augment it and work as scaffolds for tendon regeneration.

This expert opinion will explore the role of these new “bioinductive” synthetic membranes in the setting of rotator cuff surgery, focusing on their composition, the surgical technique, the clinical data available and the complications associated with these new augmentation alternatives.

## THE NEW SYNTHETIC MEMBRANES FOR ROTATOR CUFF AUGMENTATION

Although there is intense basic science research on the use of different membranes for RCR supplementation, currently there are only four options commercially available: the Regeneten® bioinductive implant (Smith & Nephew), a type‐1 bovine collagen membrane which has been used successfully in RCTs surgery, showing promising postoperative outcomes [38] and is available almost worldwide; the Biobrace® implant (Conmed); the Tapestry® implant (Zimmer) and the Integrity® implant (Anika therapeutics). These latter three are not currently available in Europe and have very limited evidence of their efficacy in augmenting RCRs. Table 1 summarises the main differences between the implants.

**Table 1:** Summary of the main characteristics the implants.

IMPLANT (Manufacturer)	Composition	Measures	Fixation	Time required to implant the scaffold	Number of studies (number of subjects)
Regeneten® (Smith & Nephew)	Purified type‐I bovine collagen	Thickness: 1 mm Sizes: 30 × 20 mm and 20 × 24 mm	PLLA staples to tendon tissue PEEK staples to greater tuberosity	14 min	27 (995)
Biobrace® (ConMed)	Highly porous type I bovine collagen + bio‐resorbable PLLA	Thickness: 3 mm Size: 23 × 30	4 sutures to the tendon 2 knotless anchors to the greater tuberosity	No information available	1 (49)
Tapestry® (Zimmer Biomet)	70% of PLLA + 30% purified type‐I collagen of bovine origin	Thickness: 1.5 mm Sizes: 26 × 26 mm and 30 × 30 mm	Absorbable staples to the tendon Absorbable staples to the bone	5 min	0 (0)
Integrity® (Anika Therapeutics)	80% Hiaff + 20% PET	Thickness: 1 mm Sizes: 20 × 25 mm and 25 × 30 mm	Absorbable PLGA staples to the tendon PEEK staples to the greater tuberosity	No information available	0 (0)

*Note*: Number of studies published in journals indexed in Pubmed (aggregated number of patients in which the implant was used in those studies). Hiaff (a proprietary chemically modified hyaluronic acid).

Abbreviations: PEEK, polyether ether ketone; PET polyethylene terephthalate; PLGA, poly‐lactic‐co‐glycolic acid; PPLA, poly L‐Lactide.

### The Regeneten bioinductive implant (Regeneten®) (Figure 1)

**Figure 1:**
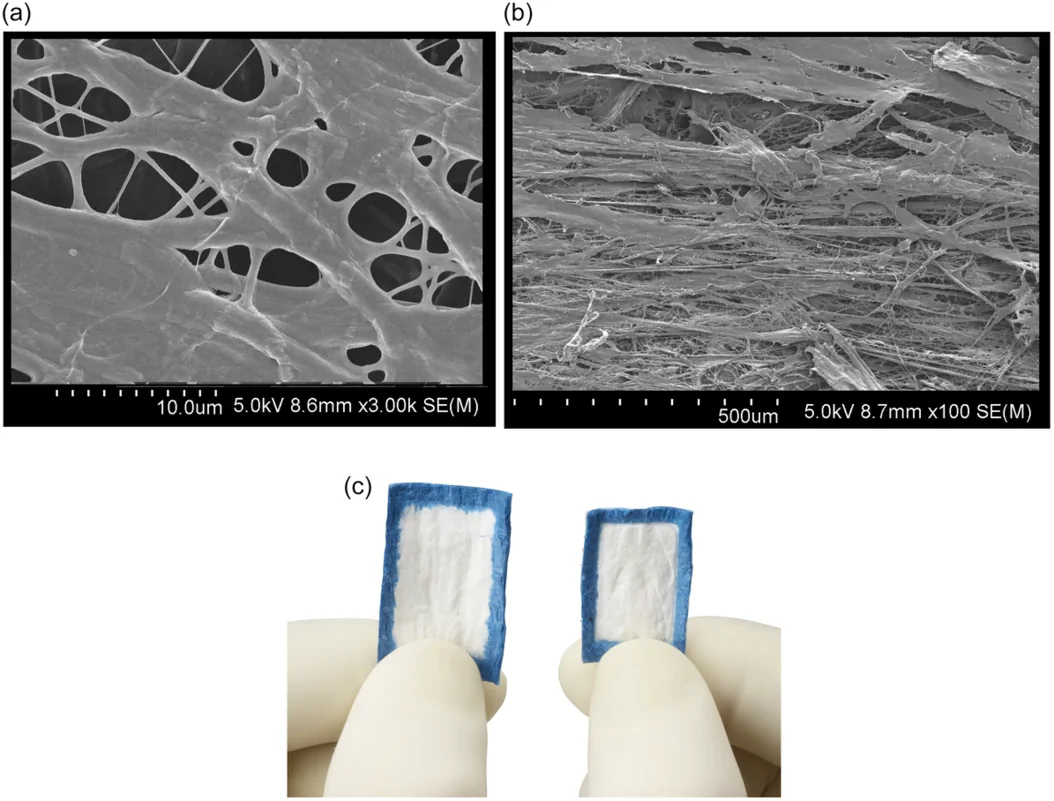
(a, b) Scanning electron microscope images showing the disposition of the collagen fibres, forming a three‐dimensional scaffold with bioinductive capacity. Lower magnification image shows the longitudinally organised structure (b), and higher magnification images show the three‐dimensional matrix (c). Images courtesy of Smith & Nephew Inc. (C) The regeneten collagen bioinductive implant in the large size available.

Regeneten® is a bioinductive membrane composed of purified type‐I collagen derived from bovine Achilles tendons, which is denatured and reassembled into a three‐dimensional scaffold (Figure 1). Rotation Medical led the initial development of the product during the early 2000s, collaborating with a team of engineers and an Australian shoulder surgeon, Desmond Bokor. The first human implantation was performed in Australia in 2012, followed by the product's launch in the US market in 2014. It received the European Union approval in 2020, 3 years after the acquisition of the original company by Smith & Nephew, in 2017.

The membrane is a 1‐mm thick matrix, available sterile in two sizes −30 × 20 and 20 × 24 mm (Figure 1c) and is delivered within a sterile arthroscopic set of disposable instruments designed for its placement over the rotator cuff. It is securely fixed using absorbable poly L‐Lactide (PLLA) staples medially into tendon tissue and polyether ether ketone (PEEK) staples to anchor it firmly into the greater tuberosity of the humerus [38].

Initial animal studies [46] demonstrated that when the implant was placed over healthy sheep infraspinatus tendon, it increased tendon thickness by being colonised by local tenoblasts from the underlying tendon. Over time, the implant is populated by these cells, progressively reabsorbed and replaced by mature tendon‐like tissue. Subsequent histological studies in humans support these findings, showing that the implant promotes tissue regeneration by facilitating new connective tissue formation within 12–24 weeks, with complete scaffold resorption occurring by 26 weeks [2, 10, 46].

### The Biobrace implant (Biobrace®) (Figure 2)

**Figure 2:**
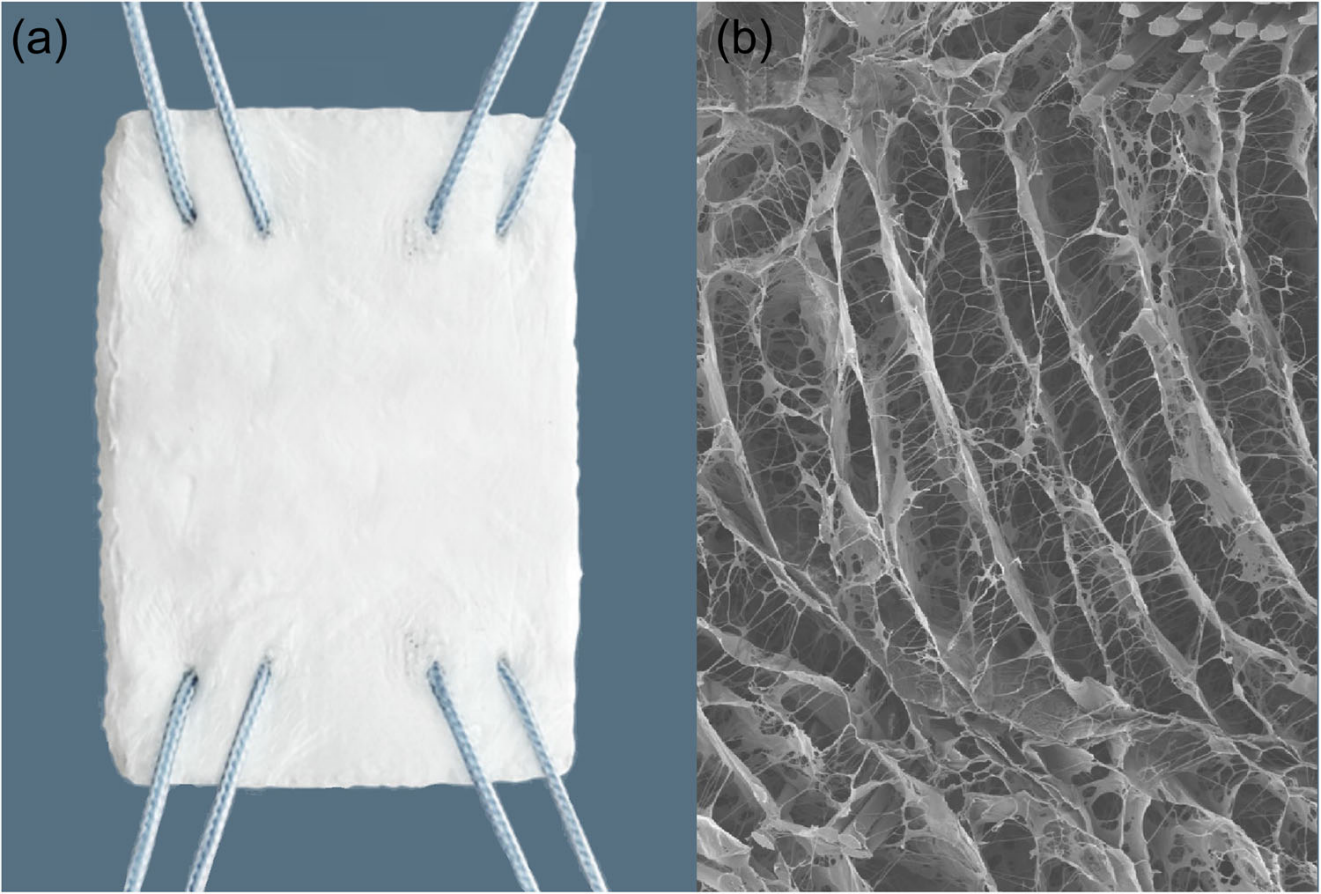
(a) The Biobrace® membrane is composed of highly porous type I bovine collagen, and bio‐resorbable poly‐L‐Lactide (PLLA) micro filaments that can be sutured to the rotator cuff tendon. (b) Scanning electron images that show the three‐dimensional structure of the membrane that facilitates tissue ingrowth. Images courtesy of Linvatec Inc.

Biobrace® is a membrane composed of highly porous type I bovine collagen, and bio‐resorbable poly PLLA micro filaments, intended for the augmentation of tendon and ligament repair. Its open 3‐D structure (Figure 2) allows for induction, maturation and remodelling of new host tissue while providing load sharing strength at the time of implantation. The membrane is a 3‐mm‐thick matrix that is presented sterile in one size, 23 × 30 mm. As it has mechanical properties similar to a native tendon [47], and designed to be sutured over the affected cuff tendon in a procedure that can be performed arthroscopically [13].

Unpublished data suggest that it incites a robust native healing response and the formation of regularly oriented connective tissue fibres in large animal models [30].

### The Tapestry biointegrative implant (Tapestry®) (Figure 3)

**Figure 3:**
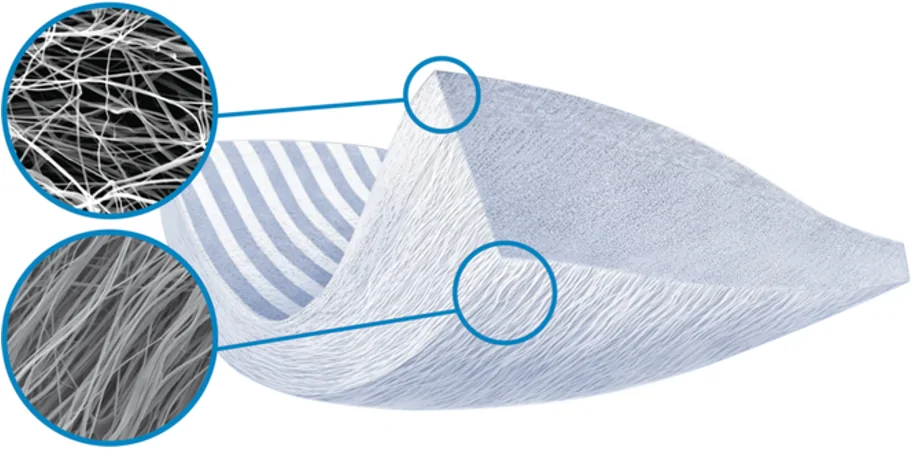
The Tapestry® matrix is composed of 70% poly L‐lactide and 30% purified type‐I collagen of bovine origin. It has highly aligned and highly porous structure with consistent microarchitecture similar to native tendon and gradually resorbs leaving new tendon‐like tissue in place of the implant. Images courtesy of Zimmer Inc.

Tapestry® is a matrix composed of 70% of PLLA and 30% purified type‐I collagen of bovine origin [26]. It has highly aligned and porous structure with consistent microarchitecture similar to native tendon and gradually resorbs leaving new tendon‐like tissue in place of the implant. The product was developed by Embody Incorporated and was approved by the FDA in 2020, listing the Regeneten® bioinductive implant as the predicate device for approval. It has not been approved for use in the EU. The membrane is a 1.5 mm thick matrix that is presented sterile in two sizes, 26 × 26 and 30 × 30 mm (Figure 3), in conjunction with a set of arthroscopic disposable instruments that allow the matrix to be placed over the rotator cuff and fixed securely with absorbable staples (used to fix it into tendon tissue and into the greater tuberosity bone). There is no published evidence on the safety and efficacy of the membrane for the management of RCR, either in the basic science or clinical setting.

### The Integrity implant (Integrity®) (Figure 4)

**Figure 4:**
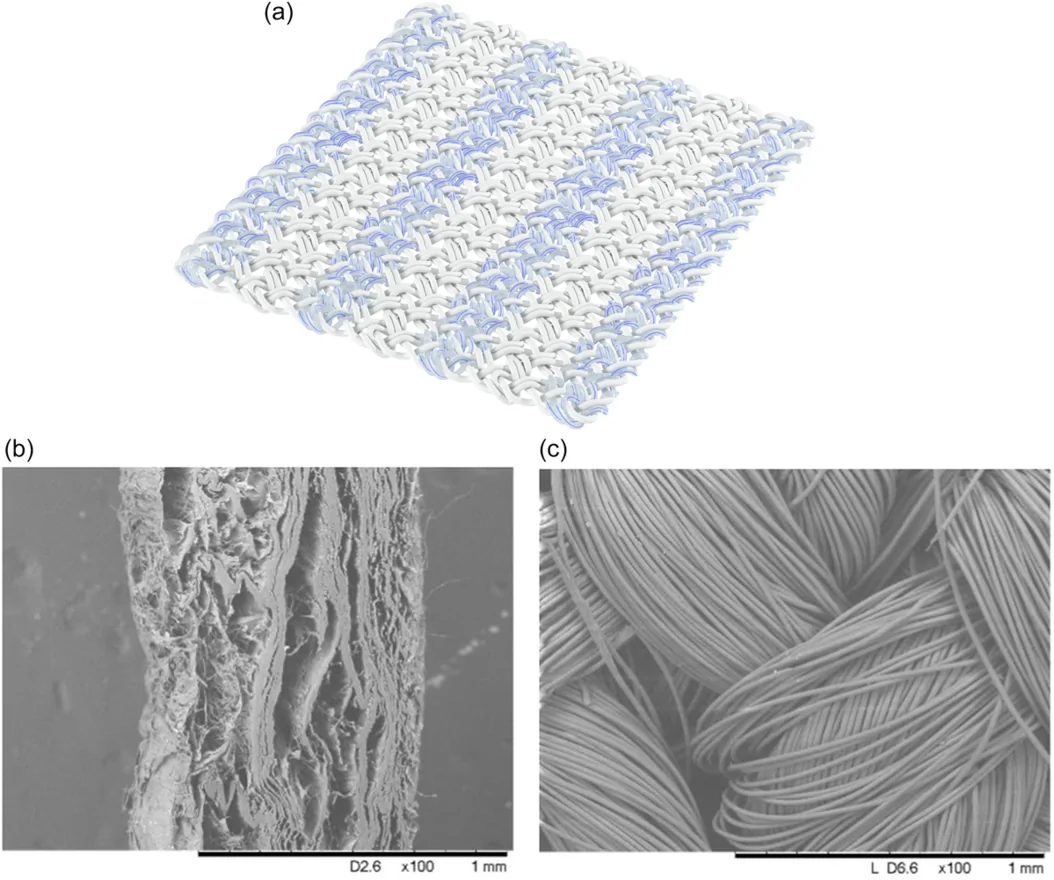
(a) Image of the Integrity® bioabsorbable scaffold, which is composed of 80% chemically modified hyaluronic acid (Hiaff) and 20% polyethylene terephthalate (PET). (b, c) Scanning electron imaging allows to see the 1 mm thickness of the membrane and the carefully interwoven fibres. Images courtesy of Anika Inc.

Integrity® is a bioabsorbable scaffold composed of 80% chemically modified hyaluronic acid (Hiaff) and 20% polyethylene terephthalate (PET). Hiaff is a benzyl‐ester derivative form of hyaluronic acid (HA), that exhibits superior stability and strength as well as a slower rate of degradation compared to unmodified HA and degrades into benzyl alcohol and HA. The PET gives the construct strength, biostability and support of tissue growth. Its application in tendon repair provides a temporary matrix that supports fibroblast infiltration and collagen deposition while minimising local inflammation. The scaffold is gradually resorbed over approximately 3–6 months, allowing native tendon tissue to remodel and mature while leaving minimal residual PET once Hiaff is resorbed. The membrane is a 1‐mm thick matrix that is presented sterile in two sizes, 20 × 25 mm and 25 × 30 mm, in conjunction with a set of arthroscopic disposable instruments for placing the matrix over the rotator cuff and fix it securely with a combination of absorbable poly‐lactic‐co‐glycolic acid (PLGA) staples to fix it medially into the tendon and PEEK staples to anchor it into the greater tuberosity. There is very limited evidence, based on white papers provided by the manufacturer, on the safety and efficacy of the membrane for the management of RCR, either in the basic science [3] or clinical setting [35].

## SURGICAL TECHNIQUE

In all four membranes, the surgical procedure is traditionally performed in an all‐arthroscopic fashion, with the Regeneten®, Biobrace®, Tapestry® and Integrity® implants providing specific instrumentation to facilitate all‐arthroscopic placement and fixation. The membranes can also be used during an open or mini‐open RCR.

The reported time required for scaffold application is approximately 14 min for Regeneten® [36] and about 5 min for Tapestry® (manufacturer technical note). No published data are available regarding the surgical application time for Biobrace® or Integrity®.

Detailed animations of the implantation procedures, provided by the manufacturers, are available here:

– Regeneten®: https://youtu.be/xVEjxAjfFMQ?si=d279WYarvvgRjUsQ
– Biobrace®: https://youtu.be/YT56WUitQKg?si=yPUJU74ml9dykqxj
– Tapestry®. https://www.zimmerbiomet.com/en/products-and-solutions/specialties/sports-medicine/tapestry-rc-biointegrative-implant-system.html
– Integrity®: https://youtu.be/zSjpagSeRos?si=UhcOBpoUPjkK29eN

### Synthetic membrane implantation over a full‐thickness posterosuperior cuff repair (Figures 5 and 6)

**Figure 5:**
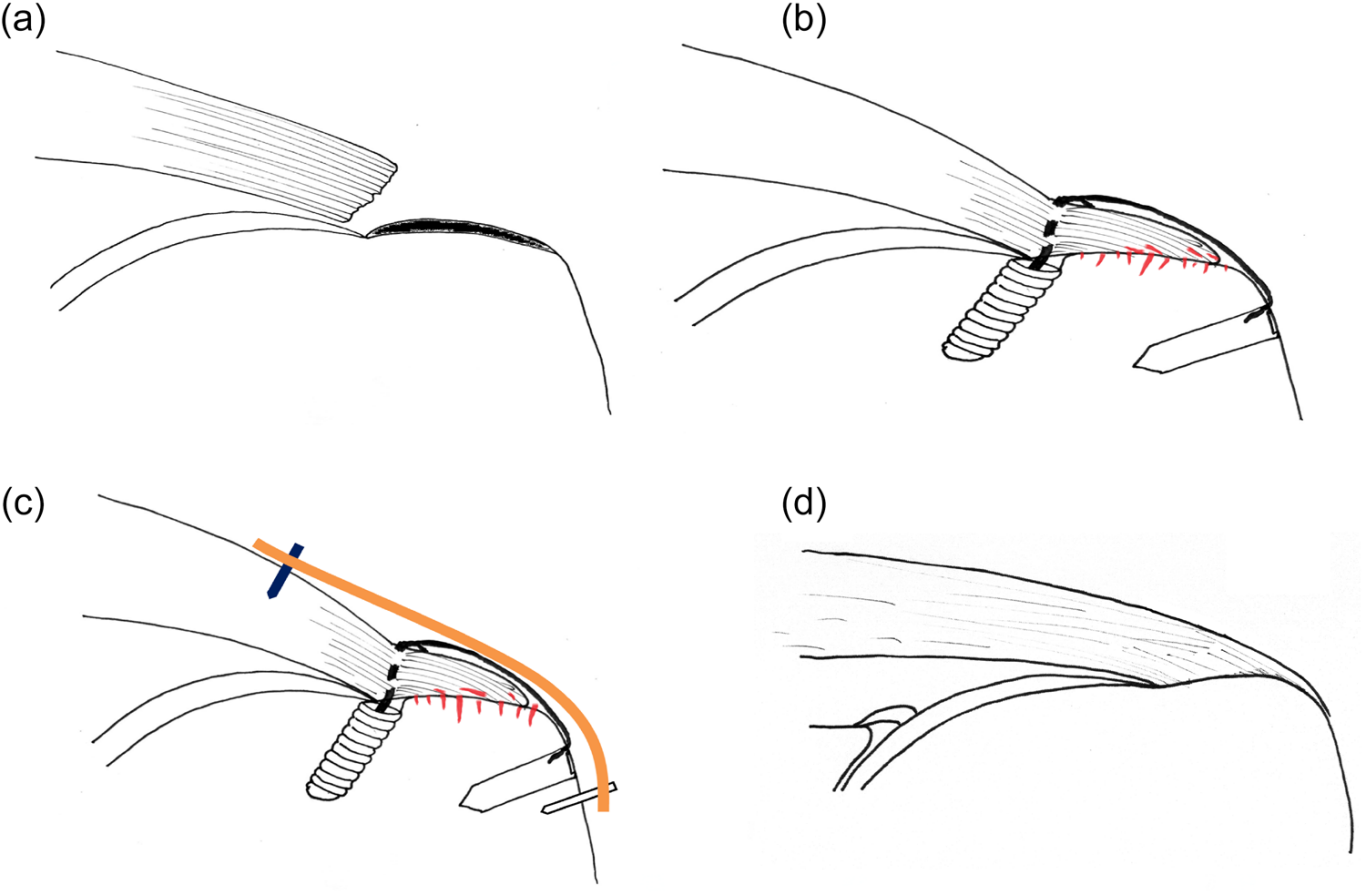
Schematic diagram showing the use and the effect of a bioinductive collagen implant (Regeneten) as a supplement of full‐thickness rotator cuff repair (RCR). (a) The tear (b) RCR in a transosseous equivalent (TOE) configuration. (c) Placement of the Regeneten (orange) over the RCR with medial anchors (deep blue) and lateral anchors (grey). (d) Healing of the tendon, with complete resorption of the Regeneten and the footprint of the new tendon extending slightly over the tuberosity.

**Figure 6:**
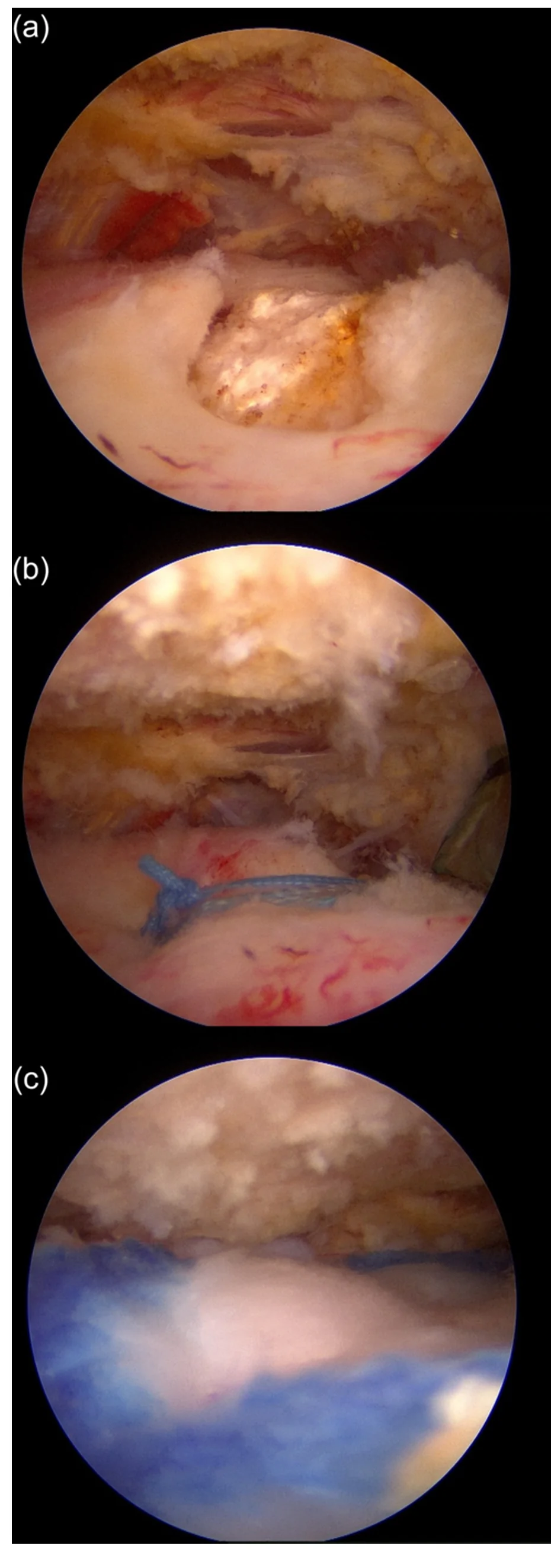
Arthroscopic images from the posterior viewing portal of a right shoulder showing the use and the effect of a bioinductive collagen implant (Regeneten) as a supplement of a right full‐thickness posterosuperior rotator cuff repair. The repair is assessed first (a), then repaired using a transosseous technique (b) and the membrane is placed over the repair and fixed to the tendon and lateral bone (c).

First, the tear and the long head of the biceps should be evaluated from either the articular or bursal side, after which a decision should be made regarding a tenotomy, tenodesis or preservation. The FT‐RCT should be repaired using a standard arthroscopic technique—either single‐row, double‐row or transosseous equivalent (TOE) repair—based on the surgeon's preference. The lateral portal for cuff repair should be developed parallel to the tendon footprint, taking into consideration the subsequent implantation of the membrane, thereby facilitating its introduction. Alternatively, a more superior portal may be established initially, with an accessory lateral and distal portal created later to allow for precise placement of the membrane into the subacromial space.

Following RCR, the target area for membrane placement over the repaired tendon is identified. In most cases, the membrane is placed into the subacromial space through the lateral portal. For larger of more complex tears, the surgeon must weigh the chances of healing of specific regions of the repair and the functional importance of the involved tendons. Generally, the portion of the repair at higher risk of failure should be prioritised for membrane‐coverage. However, in massive tears with extensive risk zones, the posterosuperior aspect of the repair is often selected for protection with the membrane. At this stage, the accessory cannulas used for medial fixation are typically introduced into the subacromial space, as they assist in achieving appropriate orientation and positioning of the membrane over the repaired tendon.

The membrane is then placed into the subacromial space and positioned over the bursal surface of the repaired tendon, extending approximately 5 mm lateral to the edge of the footprint. Fixation is performed, depending on the specific implant system, either from medial to lateral (Regeneten®, Tapestry® and Biobrace®) or from lateral to medial (Integrity®) using the specific anchors provided by the manufacturers. The implant should be secured under slight tension to ensure intimate contact with the underlying tendon.

### Synthetic membrane implantation over a partial‐thickness rotator cuff tear (PT‐RTC) (Figures 7 and 8)

**Figure 7:**
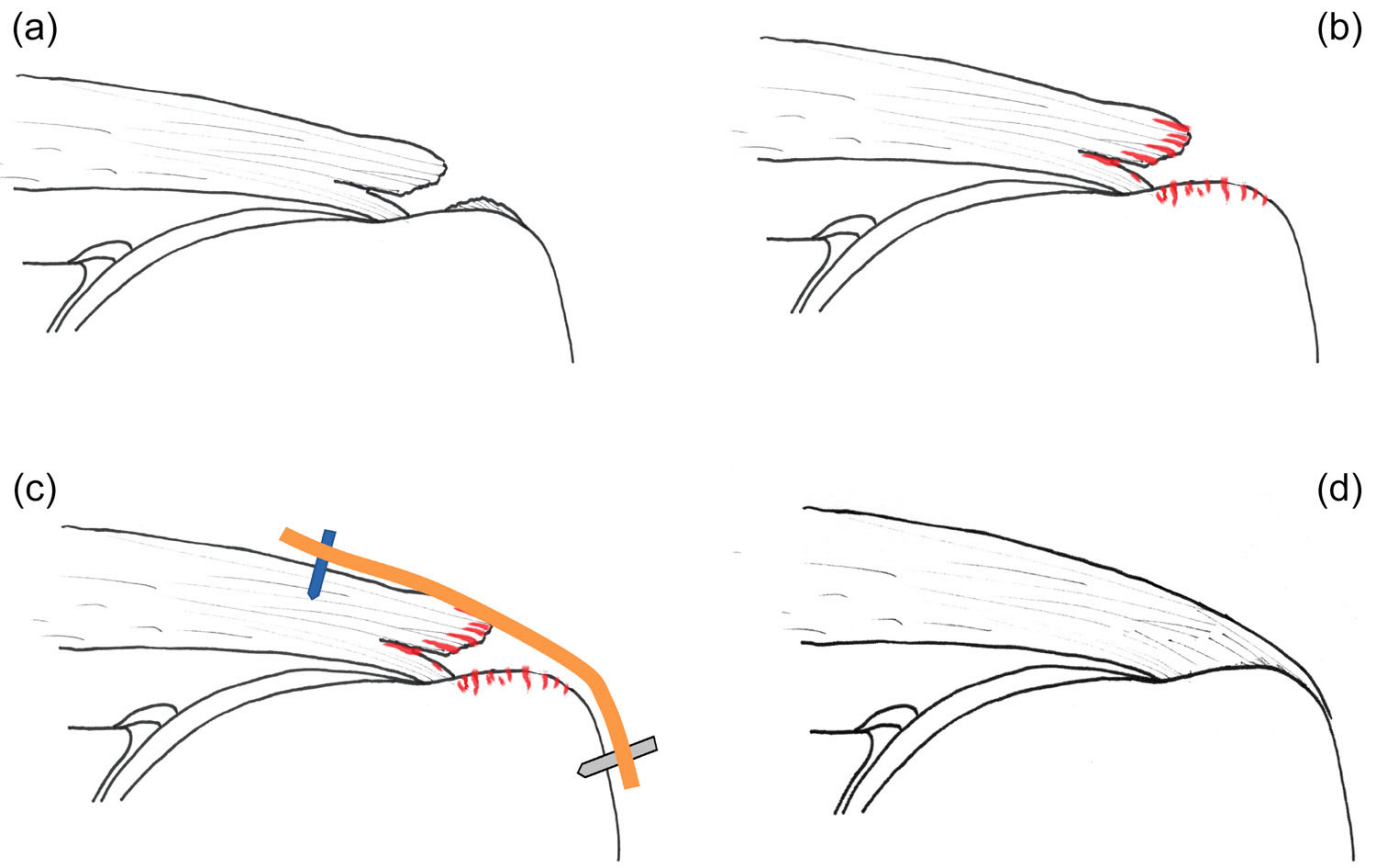
Schematic image showing the use and the effect of a bioinductive collagen implant (Regeneten) used as an isolated treatment for a bursal‐sided partial‐thickness rotator cuff tear (PT‐RCT). (a) The bursal‐sided PT‐RCT. (b) The tear is debrided. (c) Placement of the Regeneten (orange) over the rotator cuff repair (RCR) with medial anchors (deep blue) and lateral anchors (grey). (d) Healing of the tendon, with complete resorption of the Regeneten and the footprint of the new tendon extending slightly over the tuberosity.

**Figure 8:**
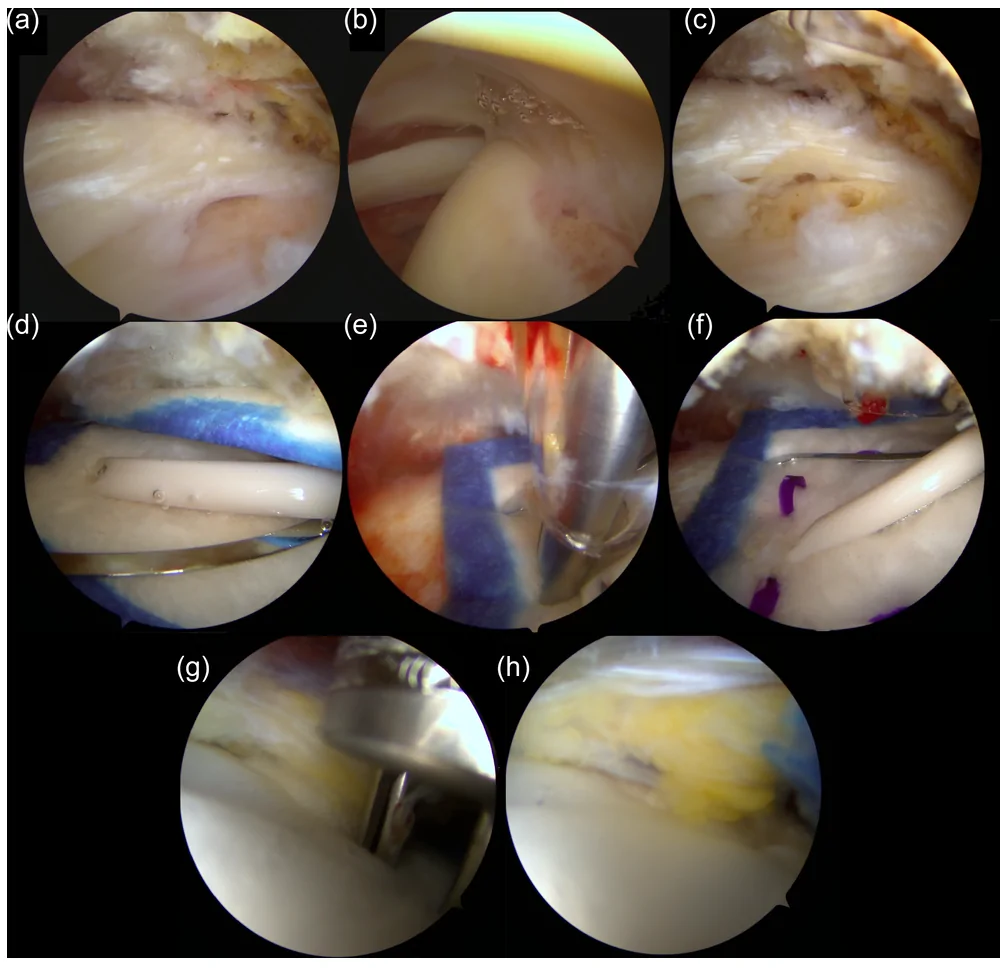
Arthroscopic image from the posterior portal of the use of a bioinductive collagen implant (Regeneten) as isolated treatment for a left bursal‐sided partial‐thickness rotator cuff tear (PT‐RCT). The tear is identified form the subacromial space (a), integrity of the tendon is confirmed form the articular side (b). The bursal tear is debrided and the exposed footprint microfractured (c). The Regeneten is placed into the subacromial space form the lateral portal with the help of the insertion device (d), extended over the tear and then fixed medially with absorbable anchors (e, f) and laterally with polyether ether ketone anchors (g, h).

In cases of articular PT‐RCTs, the extent of the tear is assessed from the articular side, followed by proper debridement to ensure the existence of an intact and mechanically viable bursal layer. For bursal PT‐RCTs, the integrity of the articular surface of the tendon is verified arthroscopically from the articular side and careful debridement of the bursal lesion is essential to confirm the presence of a structurally competent articular layer. The long head of biceps tendon must also be evaluated, and a decision should be made regarding to perform tenotomy, tenodesis or preserving the tendon intact based on intraoperative finding and patient‐specific factors.

The following step is defining the position of the cuff defect, or the biceps tendon if left intact, in the subacromial space with the help of a couple of free needles introduced percutaneously through the tendon under direct arthroscopic visualisation of the articular side. Then, a bursectomy is performed in the subacromial space to allow clear visualisation of the bursal tear and/or the tendon markers. After that, the membrane implantation proceeds according to the steps previously described for FT‐RCT.

### Postoperative rehabilitation protocol

Postoperative rehabilitation protocols are determined based on the specific surgical procedure performed. For membrane‐augmented FT‐RCTs, rehabilitation should follow the standard practice established at each institution for full‐thickness RCRs [38]. In contrast, patients treated for PT‐RCTs using membrane augmentation alone may follow a more accelerated rehabilitation protocol. In these cases, sling immobilisation can typically be limited to 10–14 days or less, as the surgical intervention is less invasive and associated with lower postoperative pain levels. Active‐assisted range of motion exercises may begin after the first week. Once full range of motion is restored, strengthening exercises can commence as early as the fourth postoperative week. However, return to strenuous activities should be postponed for a minimum of 12 weeks.

## OUTCOMES OF THE NEW BIOLOGICAL MEMBRANES FOR ROTATOR CUFF AUGMENTATION

As outlined below, the clinical evidence supporting the use of collagen/PLLA or hyaluronic acid‐based membranes remains limited and primarily restricted to augmentation of full‐thickness tear repairs. This stands in contrast to the Regeneten® implant, for which relatively robust clinical data are available in the literature. The primary indications for Regeneten® include augmentation of full‐thickness RCR and treatment of PT‐RCTs without repair. For a more comprehensive overview, two recent systematic reviews summarise the current body of evidence on this topic [21, 24].

Other potential applications, such as placement of the membranes over an intact but tendinopathic tendon, augmentation of an isolated subscapularis repair [1], reinforcement of the subscapularis repair following tenotomy in total shoulder arthroplasty [5], or application over FT‐RCTs without concomitant repair [11], are supported by only limited clinical evidence and are not discussed in detail in this review.

### The new synthetic membranes for augmentation as isolated treatment of partial thickness posterosuperior tears

Currently, there is no clinical or basic science data supporting the use of the Integrity®, Tapestry® or Biobrace® implants as standalone treatment for the management of PT‐RCTs.

**Figure 9:**
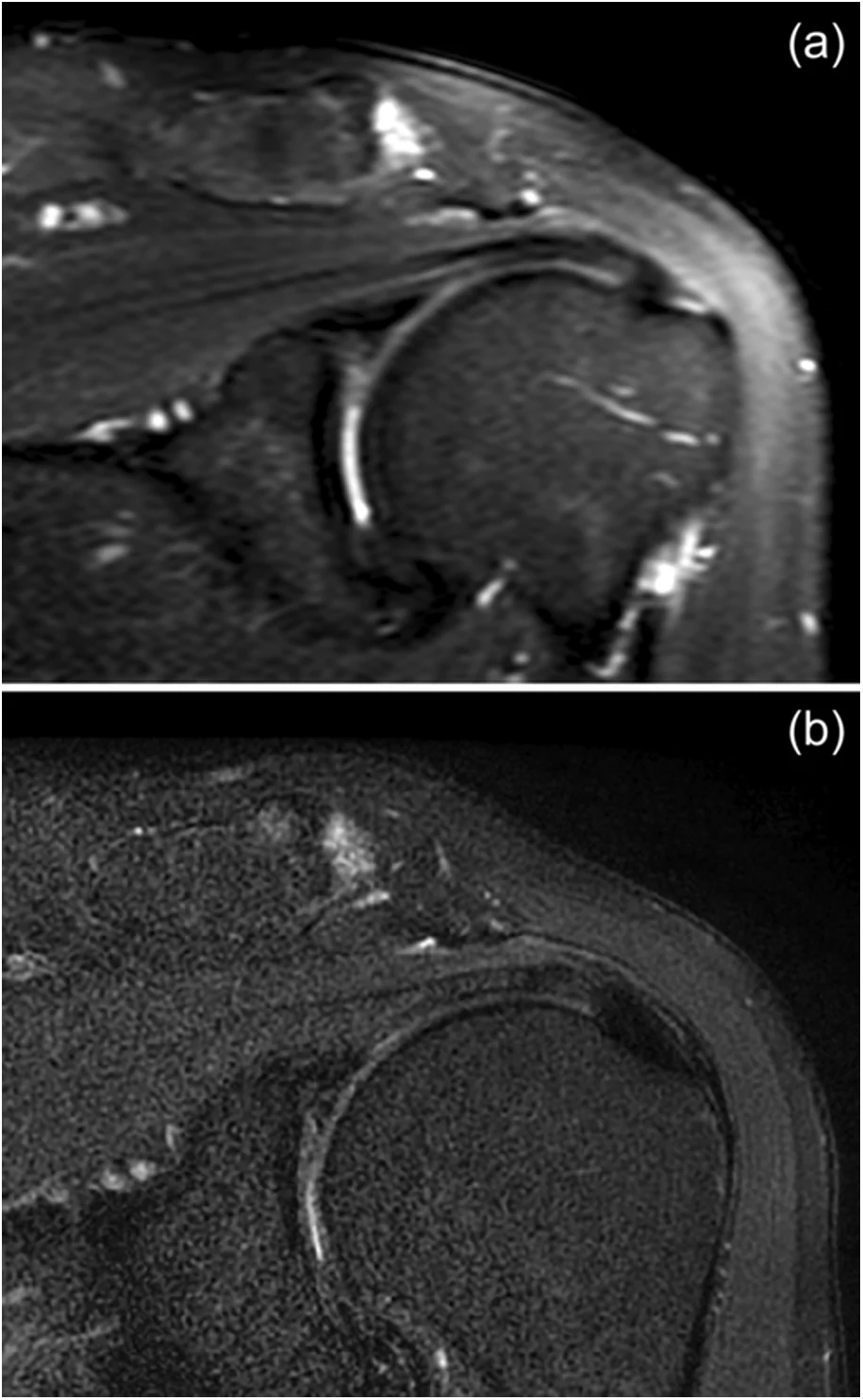
(a) Magnetic resonance image (MRI; coronal view T2) that shows a high‐grade partial bursal supraspinatus tear. (b) MRI image of the same patient 1 year after isolated Regeneten® implantation over the tear, showing complete healing of the tear and additional tendinous tissue healing up to the lateral greater tuberosity.

The rationale for using Regeneten® implant in PT‐RCT is that the matrix is placed over the partially torn tear, provided the intact layer has enough mechanical integrity, without the need for further intervention on the damaged portion of the tendon. The expectation is that the Regeneten® will be infiltrated by tenoblasts and progressively remodelled into an additional layer of healthy tendon tissue. This may support the healing process of bursal, articular and intratendinous partial tears by offloading stress and promoting regeneration in the affected regions of the tendon (Figure 9) [15].

In a pioneering 2016 study, Bokor et al. analysed the efficacy of the isolated implantation of the Regeneten® patch over a PT‐RCT for the first time [7]. They evaluated the effect of isolated Regeneten® implantation in 13 patients with intermediate‐ and high‐grade PT‐RCTs. At the 24‐month follow‐up, patients showed significant clinical improvements, including increases in the Constant score, Constant pain score, ASES total score and ASES pain score. Additionally, a significant increase in tendon thickness was observed at 3 and 12 months postoperatively. By this time, most patients exhibited tendons that were nearly indistinguishable from native tissue, although a slight decrease in thickness was noted at 24 months. Despite this reduction, tendon thickness remained greater than preoperative levels. Regarding tear healing, 7 out of 13 patients demonstrated complete healing by 12 months, three exhibited partial healing (>50%), and in the remaining three, the tear could not be identified, although MRI demonstrated progressive improvement in tendon quality. Notably, none of the 13 patients experienced tear progression during the 24‐month follow‐up. The same authors reviewed these cases at 5 years follow‐up [8], with 11 patients still available. The clinical improvements were sustained, with scores remaining significantly higher than preoperative values. MRI showed a decrease in tendon thickness compared to earlier follow‐ups, but the tendons were still thicker than before the surgery. Complete healing was observed in 8 of the 11 patients. Two patients developed a new low‐grade tear near to the previous healed site, and one patient developed a new intrasubstance delamination.

Dai et al. [16] treated 24 consecutive patients with PT‐RCTs involving more than 50% tendon thickness using isolated Regeneten® implantation. The ASES and VAS scores improved significantly from 45.6 preoperatively to 68.1 postoperatively (*p* = 0.001), and from 8.3 to 3.8 (*p* < 0.001), respectively at a mean follow‐up of 19.1 months. A significant increase in tendon thickness was also observed, from 5.7 mm preoperatively to 6.5 mm at a mean follow‐up of 9.9 months (*p* = 0.007). Most patients reported satisfaction with the procedure, with an average satisfaction score of 7.5 out of 10.

Likewise, Schlegel et al. [39] analysed the outcomes of 33 patients treated with isolated implantation of the Regeneten® membrane. Twelve patients had intermediate‐grade (25%–50%) tears, and 21 patients had high grade (>50%) tears. Clinically, 80.6% of the patients reported satisfaction with their outcomes. MRI showed complete healing in 18% of cases and a reduction in tear size in 76% of patients, with tendon thickness increasing from 3.1 mm preoperatively to 5.4 mm at 3 months. This thickness remained stable at 12 months: 24% of tears were no longer visible and 70% demonstrated further size reduction. The newly formed tissue over the tendon was indistinguishable from the native tendon by 12 months. Both articular‐ and bursal‐sided tears showed significant improvements, with 91% and 90% of these tears, respectively, either fully healed or reduced in size. In a subsequent study, the same authors [39] reported progression to a FT‐RCT in only one noncompliant patient. At the 2‐year follow‐up, new tissue filled the original tear in all patients with intermediate‐grade tears and in 95% of those with high‐grade tears. Defect fill‐in of at least 50% was observed in 90.9% of intermediate‐grade tears and 84.2% of high‐grade tears. Tendon thickness also increased significantly, with an average gain of 1.2 mm in intermediate‐grade tears and 1.8 mm in high‐grade tears. Overall, 86.7% of patients showed tendon thickness greater than baseline.

Recently, Doyle et al. [17] retrospectively reviewed subjects with high‐grade (Ellmann 3) partial‐thickness posterosuperior tear treated at their institution with at least 12 months follow‐up that underwent either isolated Regeneten® implantation or completion of the tear and repair. They did not find relevant clinical differences at 12 months follow‐up with a rate of revision repair at final follow‐up 2.1% versus 5.4% (*p* = 0.577).

Thus, the available evidence on Regeneten® for PT‐RCTs is currently limited to case series and retrospective comparative studies, considered to be of relatively low quality. However, at least two multicenter randomised controlled trials (RCT) are currently underway. One, led by Dr. Allan Wong, in Australia, has completed patient recruitment and is expected to publish results in 2025. The other is a large RCT sponsored by the manufacturer, comparing isolated Regeneten® placement with complete‐and‐repair for high‐grade PT‐RCTs (Clinical trials ID: NCT05444465).

### The new synthetic membranes as augmentation of a repair for full thickness posterosuperior tears

The principle supporting the use of these novel membranes is the promotion of new tendinous tissue formation, incorporated from the membrane placed over the repaired tendon following surgical repair. This newly formed tissue is intended to support the healing process and enhance the quality of the degenerative tendon, thereby reducing the risk of both failures at the tendon‐to‐bone interface and the occurrence of medial retears adjacent to the repair construct. Currently, there is no published evidence that supports the use of the Tapestry® implant for this indication.

#### Regeneten® evidence

Bokor et al. were, again, the first to report on the use of Regeneten® as an adjunct to the repair of a FT‐RCT [7]. They analysed outcomes in eight patients with medium‐sized FT‐RCTs who underwent various types of tendon repair, followed by the application of the Regeneten® implant. Significant improvements were observed in both Constant (from 50.7 to 78; *p* < 0.001) and ASES scores (from 44.6 to 87.8; *p* < 0.001) scores. At 24 months follow‐up, 89% of patients reported satisfaction with the outcome. A significant increase in tendon thickness was also noted at 3 months postoperatively, which remained stable at the 12‐month follow‐up. Although a slight reduction of the tendon thickness was observed at 24 months, it remained greater than preoperative values.

Bushnell et al. [9] arthroscopically augmented 115 medium to large FT‐RCTs with Regeneten® in a prospective multicentric study. Clinical improvements were observed in more than 90% of cases, and healing was observed in MRI in 95% of medium sized tears and 65% in larger tears at 2‐year follow‐up. Two major complications were reported: one case of deep infection and one case of persistent inflammation.

**Figure 10:**
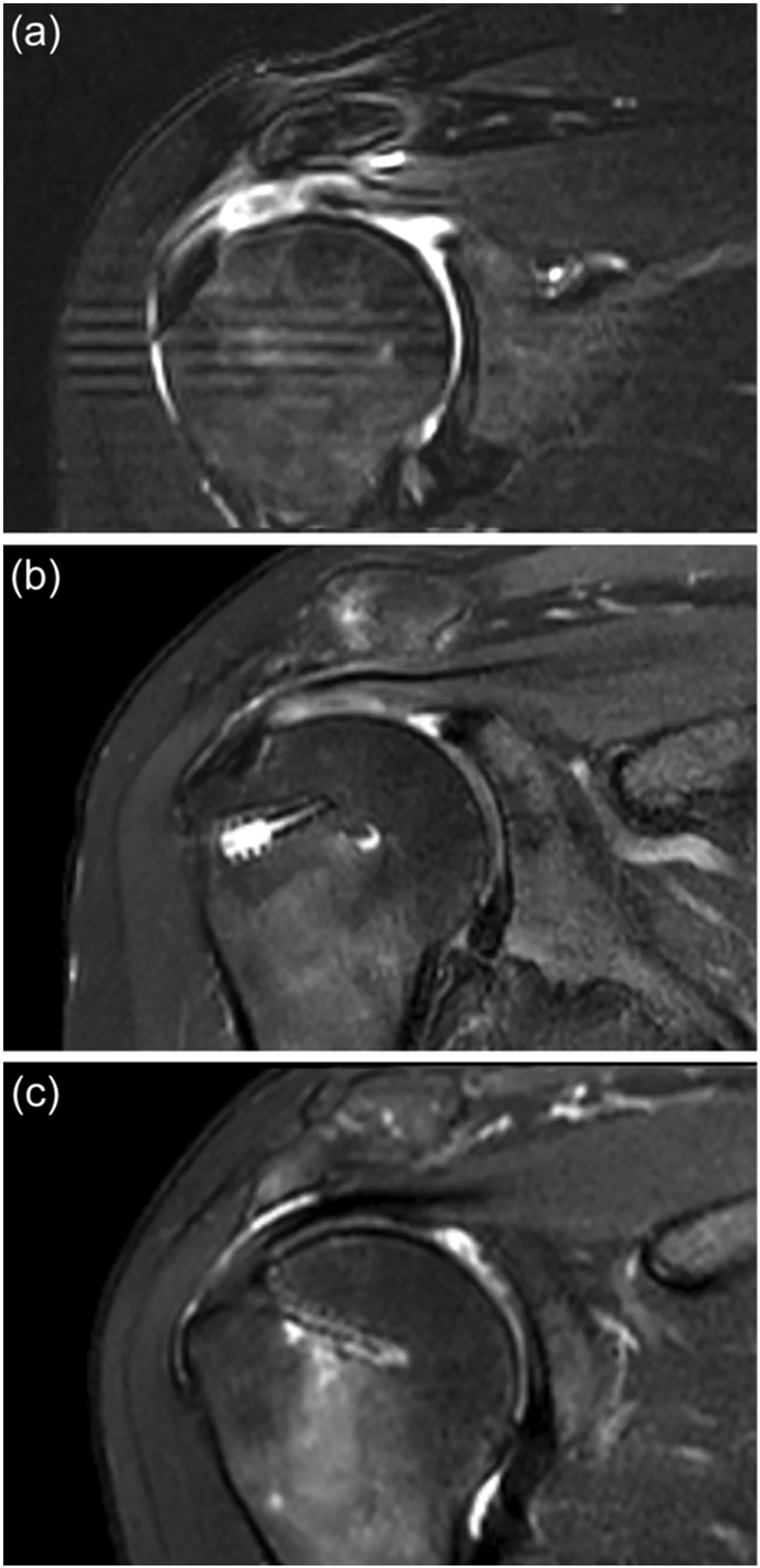
(a) Magnetic resonance image (MRI; coronal view T2) that shows a full‐thicnkess supraspinatus tear. (b) MRI image of the same patient 1 year after transosseous repair followed by Regeneten® implantation over the tear, showing healing of the tear with mild degenerative changes at the repair site. (c) MRI image of the same patient 2 years showing a healed tendon.

Ruiz Iban et al. [37] conducted a 2‐arm randomised study comparing a TOE repair with and without the addition of Regeneten® in 124 patients with small to medium size tears (1–4 cm). They performed clinical and radiological assessment of these patients at both 1‐ and 2‐year follow‐up and an extension of the study to 5‐year outcomes is underway. After a mean follow‐up of 25.4 months, the study found no significant differences between the groups in achieving the minimal clinically important difference (MCID) for the ASES and Constant‐Murley scores. However, in MRI performed at 2‐year follow‐up, the Regeneten group showed a significant decrease in retear rate (12% in the Regeneten group vs. 35% in the control group, *p* = 0.004; Figure 10). Also, a post hoc analysis of the clinical data showed that the presence of a retear in MRI at 2‐year follow‐up was associated with poorer outcomes as subjects with an intact tendon presented better Constant‐Murley Score values (*p* = 0.035), ASES scores (*p* = 0.015) and pain scores (*p* = 0.006) than those with a failed repair.

In contrast with these findings, Haft et al. [20] made a retrospective analysis of 47 subjects with medium to large posterosuperior tears repaired and supplemented with a Regeneten® implant. They retrospectively matched the outcomes with a cohort of similar patients without augmentation and did not find clinical differences at 2‐year follow‐up. They did not perform any imaging analysis but found an increased reoperation rate (9% vs. 0%, *p* = 0.01) due to stiffness and inflammation.

In the context of large and massive RCTs, the available data remains limited. Zhang et al. [49] compared 24 patients with retracted large and massive tears treated with arthroscopic repair augmented with Regeneten® to 24 patients with similar tears treated with the repair but without augmentation. While both groups showed improvement, no significant differences were found in the clinical outcomes or in the MRI assessment. The healing rates were 55% in the Regeneten® group and 53% in the control group.

In the context of revision surgery, Thon et al. [43] evaluated outcomes in 23 patients with large to massive RCTs treated with tendon repair augmented with the Regeneten® membrane. Of these, 16 patients had undergone previous surgery, while 7 were undergoing primary repair. No significant differences in ASES scores were found between primary and revision repairs or between large and massive tears. Tendon thickness consistently increased following surgery, and healing was confirmed by both MRI and ultrasound in 96% of patients at 24 months. However, no significant differences in healing rates were observed between large and massive tears, nor between primary and revision surgeries. Regarding range of motion, Berthold et al. [6] evaluated five patients who underwent revision RCT repair augmented with Regeneten®, in combination with bursa, PRP, platelet‐poor plasma and autologous thrombin. They observed significant improvements in forward flexion (3°), abduction (10°) and external rotation (38°) at a mean follow‐up of 6.5 months. Ting et al. [44] conducted a post hoc matched cohort study in a workers’ compensation population with a history of failed RCR. They compared outcomes in 19 patients who underwent RCT with Regeneten® and 32 patients who underwent repair without augmentation. At the 6‐week follow‐up, the control group showed greater external rotation and forward elevation. Similarly, at the 3‐month follow‐up, greater flexion and forward elevation were observed in the nonaugmented group compared to the Regeneten® group. In contrast, patients in the Regeneten® group showed greater internal rotation and adduction strength at 3 months. At final follow‐up, the retear rate in the Regeneten® group was 47% at a mean 14 months, which was not significantly different from the control, with a retear rate of 38% at 29‐month follow‐up (*p* = 0.489).

#### Biobrace® evidence

McMillan et al. [31] reviewed retrospectively a case series of 49 subjects undergoing RCRs augmented with the BB Collagen/PLLA scaffold for large and massive full‐thickness posterosuperior cuff tears and found excellent healing rates at 2‐year follow‐up (94%) but a reoperation and complication rate of 8% and 14% respectively.

#### Integrity® evidence

Porter et al. [35] presented, in a manufacturer's white paper, the results of a poorly described cohort of 29 patients who underwent a RCR supplemented with the Integrity® implant. They found good clinical outcomes at 6‐month follow‐up, but four patients required steroid injections due to stiffness.

## COMPLICATIONS ASSOCIATED WITH THE NEW MEMBRANES

As can be expected, any technology is associated with possible complications. Stiffness, infection, implant migration, or foreign body reactions have been reported as possible complications related to the use of these membranes.

### Stiffness

Yeazell et al. [48], in a retrospective study, evaluated 32 patients with high‐grade PT‐RCTs of the supraspinatus treated with Regeneten®, and paired them to a historical control group. A the 3‐month follow‐up, 8 patients in the Regeneten® group (25%) demonstrated significantly higher rates of shoulder stiffness compared to only one case (3%) in the control group (*p* < 0.001). Six of these eight patients required additional surgical intervention to address the stiffness. Similarly, Haft et al. [20] retrospectively analysed outcomes in 47 partial and FT‐RCTs tears treated with the Regeneten® membrane and compared them to a matched control group of 94 patients without augmentation. In the Regeneten® group, four patients (9%) required reintervention for postoperative stiffness, whereas no such cases were reported in the control group.

However, no other studies have reported this concerning complication. Ruiz Iban et al. [38] did not observe stiffness in any of the 64 cases treated with Regeneten® that were included in their randomised controlled trial, nor was stiffness reported in the case series by Bushnell et al. [9] or among the 33 subjects treated by Schlegel et al. [39].

### Inflammation and foreign body reaction

The use of synthetic membranes that contain collagen of animal origin, such as Tapestry®, Regeneten® and Biobrace®, may lead to foreign body reactions. Barad et al. [4] reported a case of severe subacromial‐subdeltoid inflammation with the presence of rice bodies in a patient who underwent a RCR augmented with Regeneten®, requiring subsequent surgical debridement. Likewise, Bushnell et al. [9] reported inflammatory changes observed in the MRI, along with clinical symptoms, in a patient in who received Regeneten® augmentation over a RCR at the 3‐month follow‐up. This patient also required an arthroscopic debridement to alleviate the symptoms. However, the cumulative incidence of this complication in the larger studies appears to be below 1% [21, 24].

### Implant migration

Lateral displacement of the Regeneten® was reported by Ruiz Ibán et al. [38] in one case, identified on MRI at the 1‐year follow‐up as a foreign body located in the lateral aspect of the greater tuberosity. Despite this finding, the patient showed a good clinical result and successful rotator cuff healing, he did not require further intervention.

## COST‐EFFECTIVENESS OF THE BIOLOGICAL MEMBRANES

A detailed analysis of the cost effectiveness of these different biological alternatives is difficult, especially as the specific price for each membrane is difficult to ascertain and varies greatly along different health systems and countries. Despite this, some efforts have been made to define the cost‐effectiveness of the Regeneten Bioinductive implants in different countries, specifically for the management of full‐thickness RCTs. There is no data available for the other three membranes.

An initial study by McIntyre et al. in the United States found that the associated cost of using the membrane in a RCT was 2324 US$; estimating an additional 18 healed RCTs per 100 treated patients in the first year, the estimated incremental cost‐effectiveness ratio (ICER) was $13,061 per additional healed repair. These numbers were considered to be cost saving if delays in return to work were included in the analysis or with greater tear size, as well as patients at higher risk of retear [29]. Rognoni et al., using data from Italy, showed that adding Regeneten® to standard RCR increased the proportion of healed tears, with an ICER of 17,857€ per additional healed tear for the Italian National Health service [36].

## CONCLUSION

Biologic augmentation with the new synthetic membranes is a promising technology that requires further attention. The use of the Regeneten implant seems to provide functional improvements in patients with partial tears and helps to avoid retears in full‐thickness RCTs. The evidence for the other membranes is limited at best today.

## AUTHOR CONTRIBUTIONS

The main author (Miguel Ángel Ruiz Iban) and María Josefa Espejo Reina contributed to the writing of the manuscript and preparation of the tables and figures. Jose Luis Ávila Lafuente contributed to reviewing and revising the manuscript, as well as preparing the figures. The remaining authors have read, revised and approved the final version of the manuscript. All authors have read the submitted paper and it has been approved by all above mentioned authors and we believe it represents honest work.

## CONFLICT OF INTEREST STATEMENT

The senior author (Miguel Ángel Ruiz Iban) is a consultant from Smith and Nephew and has received funding for research and education related to this manuscript. The remaining authors declare no conflicts of interest.

## ETHICS STATEMENT

The authors have nothing to report.

## Data Availability

The authors have nothing to report.
